# Secular changes in height, weight and body mass index in Hong Kong Children

**DOI:** 10.1186/1471-2458-8-320

**Published:** 2008-09-21

**Authors:** Hung-Kwan So, Edmund AS Nelson, Albert M Li, Eric MC Wong, Joseph TF Lau, Georgia S Guldan, Kwok-Hang Mak, Youfa Wang, Tai-Fai Fok, Rita YT Sung

**Affiliations:** 1 Department of Paediatrics, The Chinese University of Hong Kong, Hong Kong Special Administrative Region, PR China; 2 Centre for Clinical Trials and Epidemiological Research, The Chinese University of Hong Kong, Hong Kong Special Administrative Region, PR China; 3 Department of Biochemistry and Food and Nutritional Sciences Programme, The Chinese University of Hong Kong, Hong Kong Special Administrative Region, PR China; 4 Student Health Service, Department of Health, Government of the Hong Kong Special Administrative Region, PR China; 5 Center for Human Nutrition, Department of International Health, John Hopkins Bloomberg School of Public Health, Baltimore, MD 21205, USA

## Abstract

**Background:**

Large population growth surveys of children and adolescents aged 6 to 18 y were undertaken in Hong Kong in 1963 and 1993. The global epidemic of obesity is a major public health concern. To monitor the impact of this epidemic in Hong Kong children and to identify secular changes in growth, a further growth survey was undertaken in 2005/6.

**Methods:**

Cross-sectional height and weight measurements of 14,842 children and adolescents aged 6 to 18 y from Hong Kong's 18 districts were obtained during the 2005/6 school year. Percentile curves were constructed using LMS method and sex-specific percentile values of weight-for-age, height-for-age, and BMI-for-age were compared with those data from 1963 and 1993.

**Results:**

Secular changes in height, weight and BMI were noted between 1963 and 1993 and between 1993 and 2005/6. In the latter period, greater changes were observed at younger ages, and particularly in boys. On an annual basis, the 1993–2005/6 changes were less than those during 1963–1993. Using the International Obesity Task Force cut-offs, 16.7% of children were overweight or obese in 2005/6, which was a 5.1% increase since 1993.

**Conclusion:**

These data provide policy-makers with further evidence of the secular changes in child growth and the increasing obesity epidemic among Hong Kong children.

## Background

The global epidemic of obesity is a major public health concern [1], and an increasingly recognised problem in children and adolescents [2,3]. The prevalence of overweight and obesity and obesity-related chronic diseases have increased in adults in China during the past decade [4]. Although the World Health Organization (WHO) recommends the use of body mass index (BMI) to define obesity, Hong Kong's Student Health Service (SHS) has historically used the definition of >= 120% median weight-for-height to define obesity. Routinely collected data by the SHS has shown that for the 2005/6 school year 19.4% of primary and 16.5% of secondary students were obese using this definition and the overall proportion of children defined as obese in 2005/6 had increased by 2.7% over an eight-year period (15.7% in 1997/8 to 18.4% in 2005/6) (KH Mak, Student Health Service, Department of Health, Personal Communication). Although this routinely collected SHS data has potential to monitor trends in obesity over time, there are concerns that these data may not be representative of all Hong Kong children since only 56–58% of all Hong Kong (~80% primary, ~30% secondary) school students attend the SHS and the extent and direction of any selection bias are unknown.

In 1963, a cross-sectional study of 14,686 children from 17 schools was undertaken to develop height and weight standards for Hong Kong children aged 6 to 20 y [5]. This was followed in 1993 by a Growth Survey of 25,000 children from birth to 18 y recruited from Maternal and Child Health Centres (MCHC) and schools. These data were used to develop Hong Kong's current growth charts for weight, height, weight-for-age, weight-for-height and body mass index (BMI) [6-8]. These data also formed part of the dataset that was subsequently used to develop international BMI cut-offs [9]. The current study aimed to monitor secular changes in weight, height and BMI of Hong Kong children aged 6 to 18 y, with a particular view to documenting the extent of the evolving obesity epidemic.

## Methods

### Sample size

The study population included all school children in Hong Kong aged 6 to 18 y. Children were classified into 26 half-year age groups. As body weight can be assumed to be normally distributed, the sample size for each age group was calculated in terms of the standard deviation of the 100αth centile (*c*_100α_, alpha = 0.05 for 95^th ^percentile) and the age-specific standard deviation (SD), using the sample size planning formula developed by Healy [10]. Using the figures of the 1993 growth survey as inputs and applying the above formula, a sample size of 200 boys and 200 girls for each half-year age group in the range 6 to 9 y was estimated to keep the 95% confidence interval (CI) within ± 1.5 kg. A sample size of 300 per group for those children aged 10 y or above was estimated to keep the 95% CI within ± 3 kg. Using these estimates, it was planned to recruit 14,000 children 6 to 18 y old.

### Sampling method

A two-stage cluster sampling method was used. Data from the Education and Manpower Bureau was used to compile a sampling frame of all schools in Hong Kong. International and English Schools Foundation schools, which partly cater for expatriate non-Chinese children, were excluded. In the first stage of the sampling, one primary school and one secondary school were randomly selected from each of the 18 Districts in Hong Kong. Selection of a school was based on computer-generated random numbers according to numbers of schools per district, and if the selected school declined to participate, the next randomly selected school was invited to participate. In the second stage, two classes in each grade were selected in collaboration with the school principal based on timetables and operational needs. All students of the selected classes were invited to join the study. This method allowed the data to be comparable with those obtained in the 1993 Growth Survey. Parents were informed about the study through a letter distributed by the school and asked to inform the school if they did not wish their child to participate. Parents were told that participating children would be given a record of their body measurements, and that any child with high blood pressure or other abnormal findings would be referred for further assessment. Parents were invited to complete a questionnaire providing demographic information including gestation and birth weight, and family or personal history related to risk factors for obesity. Verbal consent was obtained from the students who were then asked to completed a Self-Administered Physical Activity Checklist [11], and a locally developed and validated rapid diet assessment questionnaire. Students were asked if they had attended the SHS for a physical examination during the current or previous academic year.

### Anthropometric measurements

A team of eight trained research staff visited each selected school on a pre-arranged date to collect the anthropometric data. All instruments were validated following the standard methods of the manufacturers, and the balances were zero calibrated. Standing height without shoes was measured using a Harpenden Stadiometer (Holtain, UK) to the nearest 0.1 cm. Body weight was measured with the lightest clothing to the nearest 0.1 kg by an electronic weighing scale (Tanita BF-522, Japan).

The reported weights of the school children in the 1993 study were adjusted by subtracting the average weight (median 0.3 kg, inter-quartile range 0.3 – 0.4 kg) of the lightest school uniform in each school from the observed weight [6]. To ensure comparability of the 1993 data with our data, we used the original unadjusted measured weights from the 1993 data to compare with our unadjusted weights.

### Statistical analysis

Percentile curves were constructed using LMS method. The sex-specific percentile values (10^th^, 50^th^, and 90^th^) of weight-for-age, height-for-age, and BMI-for-age were compared with those of the 1993 data. The LMS method using maximum penalized likelihood was used to perform model fitting of the anthropometric centiles for the physical parameters [12]. The LMS method estimates the measurement centiles in terms of three age-sex-specific cubic spline curves: the *L *curve (Box-Cox power to transform the data that follow a Normal distribution), *M *curve (median) and *S *curve (coefficient of variation). In brief, if *Y*(*t*) denotes an independent positive data (e.g. height) at *t *age(year), the distribution of *Y*(*t*) can be summarized by a normally distributed SD score (*Z*) as follows:

$$Z=\frac{{\left[Y\left(t\right)/M\left(t\right)\right]}^{L\left(t\right)}-1}{L\left(t\right)S\left(t\right)}$$

Once the *L*(*t*), *M*(*t*), *and S*(*t*) have been estimated for each age *t*, the 100α th centile at *t *gestation weeks could be derived from

*C*_100*α*_(*t*) = *M*(*t*) [1 + *L*(*t*)*S*(*t*)*Z*_*α*_]^1/*L*(*t*)^

where Z_α _is the α centile of the Normal distribution (for example for the 97^th ^centile, α = 0.75 and Z_α _= 1.88).

### Ethics

The study was approved by the Joint The Chinese University of Hong Kong and New Territories East Cluster Clinical Research Ethics Committee and the Ethics Committee of the Department of Health of the Hong Kong Government.

## Results

Eighteen primary schools and 18 secondary schools, one from each of the 18 Hong Kong districts, participated in the study. Four (22%) secondary and 2 (11%) primary schools were Government schools, 12 (67%) secondary and 15 (83%) primary schools were aided schools and 2 (11%) secondary and 1 (6%) primary schools were private schools. Of all Hong Kong schools (excluding international and English Schools Foundation schools) 22% of secondary and 6% of primary are Government schools, 69% of secondary and 80% of primary are aided schools and 9% of secondary and 14% of primary are private schools. A total of 14,842 students aged 6 to 18 y from primary (n = 6916; boys = 3589 and girls = 3327) and secondary (n = 7926; boys = 3883 and girls = 4043) schools were enrolled. Seven percent (n = 520) of the primary and 10% (n = 880) of the secondary school students declined to participate in the study (15% government, 60% aided and 25% private schools). Reasons for non-participation were not recorded. Of the 85% students with valid responses, 65% (8199/12,674) (65.4% primary and 64.1% secondary) reported that they had attended the SHS during the previous 12 months.

It was possible to compare the height, weight and BMI of children over three time periods, 1963, 1993 and 2005/6, with differences evident at different ages (Tables 1,2,3) [13]. The raw dataset from the 1963 study was not available, but detailed tables provided the data for comparisons of height, weight and BMI (Tables 1, 2 and 3) and calculation of 85^th ^and 95^th ^BMI centiles (Table 4). However it was not possible to make estimates of the percentage of overweight and obese children using different cut-offs in 1963 (Table 5).

**Table 1:** Comparison of mean height (cm) of Hong Kong children and adolescents aged 6 to 18 y by gender in 1963, 1993 and 2005/6, with age groups defined as > 5.5–6.5 = 6 y

	1963			1993			2005/6						
							
Age	n	Mean	SD	n	Mean	SD	n	Mean	SD	d1	d1/yr	d2	d2/yr
**Boys**													
6	283	110.7	5.1	223	116.0	5.4	138	117.9	4.5	5.3*	0.18	1.9*	0.15
7	742	115.5	5.3	643	120.6	5.6	483	122.9	5.8	5.1*	0.17	2.2*	0.18
8	1030	120.5	5.5	724	126.1	5.4	564	127.9	5.9	5.6*	0.19	1.8*	0.14
9	1076	125.2	5.7	634	131.0	5.8	640	133.1	5.7	5.8*	0.19	2.1*	0.16
10	1144	130.0	6.1	694	136.3	5.9	602	138.5	6.5	6.3*	0.21	2.1*	0.17
11	1121	134.8	6.6	597	141.6	6.5	636	143.5	7.2	6.8*	0.23	1.9*	0.15
12	1033	140.0	7.3	638	147.8	8.0	739	151.0	8.1	7.8*	0.26	3.2*	0.25
13	1052	147.0	8.2	642	155.3	8.6	647	158.2	8.5	8.3*	0.28	3.0*	0.22
14	1288	155.6	7.8	550	162.3	7.9	672	164.2	7.6	6.7*	0.22	1.9*	0.15
15	1622	161.5	6.6	479	166.9	6.2	588	167.9	6.0	5.4*	0.18	1.0*	0.08
16	1711	164.9	5.8	430	169.5	6.5	562	170.6	5.5	4.6*	0.15	1.1*	0.08
17	1370	166.8	5.3	442	169.8	5.8	572	171.4	5.9	3.0*	0.1	1.6*	0.12
18	874	167.0	5.1	311	170.7	5.9	468	171.7	5.5	3.7*	0.12	1.0*	0.08
													
**Girls**													
6	282	110.0	4.7	211	115.4	5.5	128	117.3	5.0	5.4*	0.18	1.9*	0.15
7	742	114.7	5.1	553	119.8	5.1	488	120.8	5.3	5.1*	0.17	0.9*	0.08
8	970	120.0	5.6	647	125.2	5.6	471	127.2	5.5	5.2*	0.17	2.0*	0.15
9	1022	125.1	6.2	617	131.1	6.3	601	132.7	6.3	6.0*	0.2	1.7*	0.12
10	1178	130.5	6.7	633	137.1	6.9	554	138.4	6.8	6.6*	0.22	1.3*	0.1
11	1312	136.6	7.2	584	143.7	7.2	584	145.4	7.0	7.1*	0.24	1.7*	0.13
12	1340	144.0	7.5	579	149.6	6.7	734	151.5	6.4	5.6*	0.19	1.9*	0.15
13	1159	150.0	6.9	633	153.2	5.9	640	155.2	6.0	3.2*	0.11	2.0*	0.15
14	1152	154.0	6.1	617	156.3	5.4	667	157.0	5.3	2.3*	0.08	0.8*	0.05
15	1275	155.4	5.4	552	157.0	5.3	637	157.9	5.2	1.6*	0.05	1.0*	0.07
16	1267	155.6	5	520	157.3	5.1	590	158.4	5.4	1.7*	0.06	1.1*	0.08
17	984	155.6	4.9	536	157.8	5.3	642	158.7	5.4	2.2*	0.07	0.9*	0.07
18	614	155.6	4.7	334	158.3	5.3	453	158.7	5.7	2.7*	0.09	0.4	0.03

*: p-value < 0.05

d1: Difference of mean between 1963 and 1993

d2: Difference of mean between 1993 and 2005/6

d1/yr: Annual changes between 1963 to 1993

d2/yr: Annual changes between 1993 to 2005/6

**Table 2:** Comparison of mean weight (kg) of Hong Kong children and adolescents aged 6 to 18 y by gender in 1963, 1993 and 2006, with age groups defined as > 5.5–6.5 = 6 y

	1963			1993			2005/6						
							
Age	N	Mean	SD	n	Mean	SD	n	Mean	SD	d1	d1/yr	d2	d2/yr
Boys													
6	283	17.5	1.5	223	21.3	4.5	138	22.4	3.3	3.8*	0.13	1.1*	0.08
7	742	19.0	2.2	643	22.9	4.5	483	24.9	5.7	3.9*	0.13	2.0*	0.15
8	1030	20.6	2.6	724	25.7	5.2	564	27.7	6.4	5.1*	0.17	2.0*	0.15
9	1076	22.4	3.1	634	28.8	6.5	640	30.5	6.7	6.4*	0.21	1.7*	0.13
10	1144	24.6	3.9	694	32.4	8.0	602	35.5	9.2	7.8*	0.26	3.1*	0.24
11	1121	27.1	4.8	597	36.3	8.9	636	38.8	9.8	9.2*	0.31	2.5*	0.19
12	1033	30.0	5.9	638	39.9	10.0	739	44.4	10.8	9.9*	0.33	4.5*	0.35
13	1052	34.5	7.0	642	44.9	10.8	647	49.4	12.3	10.4*	0.35	4.5*	0.35
14	1288	40.0	6.8	550	50.5	10.5	672	53.3	11.0	10.5*	0.35	2.8*	0.22
15	1622	45.6	6.4	479	53.8	10.4	588	57.3	11.5	8.2*	0.27	3.5*	0.27
16	1711	49.0	6.0	430	57.5	9.8	562	59.5	11.2	8.5*	0.28	2.0*	0.15
17	1370	51.3	5.9	442	59.1	9.1	572	61.3	11.6	7.8*	0.26	2.2*	0.17
18	874	52.3	5.9	311	60.3	9.4	468	62.0	10.5	8.0*	0.27	1.7*	0.13
													
Girls													
6	282	16.9	1.7	211	20.2	3.5	128	21.3	4.0	3.3*	0.11	1.1*	0.08
7	742	18.3	2.3	553	22.1	4.3	488	23.0	4.7	3.8*	0.13	1.0*	0.07
8	970	20.2	2.9	647	24.9	5.1	471	26.1	5.1	4.7*	0.16	1.2*	0.09
9	1022	22.4	3.5	617	27.9	6.2	601	29.5	6.4	5.5*	0.18	1.6*	0.12
10	1178	25.0	4.2	633	31.4	7.1	554	33.2	7.8	6.4*	0.21	1.8*	0.14
11	1312	28.0	5.4	584	35.9	8.5	584	37.2	8.3	7.9*	0.26	1.3*	0.10
12	1340	32.7	6.8	579	40.3	8.7	734	42.6	9.6	7.6*	0.25	2.3*	0.18
13	1159	37.3	6.9	633	44.3	9.7	640	46.5	8.8	7.0*	0.23	2.2*	0.17
14	1152	41.4	6.3	617	47.6	8.4	667	48.4	8.5	6.2*	0.21	0.7	0.06
15	1275	43.6	5.7	552	48.7	7.0	637	49.5	8.9	5.1*	0.17	0.8	0.06
16	1267	44.5	5.3	520	49.5	7.4	590	51.1	9.1	5.0*	0.17	1.7*	0.12
17	984	45.1	5.1	536	50.5	7.2	642	51.1	9.5	5.4*	0.18	0.6	0.05
18	614	45.5	5.1	334	51.0	6.9	453	51.3	8.3	5.5*	0.18	0.4	0.02

*: p-value < 0.05

d1: Difference of mean between 1963 and 1993

d2: Difference of mean between 1993 and 2005/6

d1/yr: Annual changes between 1963 to 1993

d2/yr: Annual changes between 1993 to 2005/6

**Table 3:** Comparison of mean body mass index (BMI, kg/m^2^) of Hong Kong children and adolescents aged 6 to 18 y by gender in 1963, 1993 and 2006, with age groups defined as > 5.5–6.5 = 6 y

	1963			1993			2005/6						
							
Age	n	Mean	SD	n	Mean	SD	n	Mean	SD	d1	d1/yr	d2	d2/yr
Boys													
6	283	14.1	1.0	223	15.7	2.0	138	16.0	1.9	1.6*	0.05	0.3	0.02
7	742	14.1	1.0	643	15.7	2.3	483	16.4	2.7	1.5*	0.05	0.7*	0.05
8	1030	14.3	1.2	724	16.0	2.4	564	16.8	2.7	1.8*	0.06	0.7*	0.06
9	1076	14.9	1.1	634	16.6	2.9	640	17.1	2.8	1.8*	0.06	0.4*	0.04
10	1144	14.6	1.4	694	17.3	3.2	602	18.3	3.6	2.7*	0.09	1.0*	0.08
11	1121	14.6	1.6	597	18.0	3.4	636	18.7	3.5	3.4*	0.11	0.7*	0.05
12	1033	15.4	1.8	638	18.1	3.4	739	19.3	3.6	2.7*	0.09	1.2*	0.09
13	1052	16.1	1.9	642	18.4	3.4	647	19.5	3.7	2.4*	0.08	1.1*	0.08
14	1288	16.7	1.8	550	19.0	3.2	672	19.7	3.4	2.3*	0.08	0.6*	0.05
15	1622	17.4	1.8	479	19.3	3.3	588	20.3	3.6	1.8*	0.06	1.0*	0.08
16	1711	17.9	1.9	430	20.0	3.0	562	20.4	3.5	2.1*	0.07	0.4	0.03
17	1370	18.4	1.9	442	20.5	2.9	572	20.8	3.6	2.1*	0.07	0.4	0.02
18	874	18.7	1.8	311	20.7	2.8	468	21.0	3.3	2.0*	0.07	0.4	0.02
													
Girls													
6	282	13.8	1.0	211	15.1	1.5	128	15.4	2.0	1.3*	0.04	0.3	0.02
7	742	13.9	1.0	553	15.3	2.3	488	15.7	2.4	1.4*	0.05	0.4*	0.03
8	970	14.0	1.1	647	15.8	2.4	471	16.0	2.4	1.8*	0.06	0.3	0.02
9	1022	14.3	1.3	617	16.1	2.5	601	16.6	2.6	1.9*	0.06	0.5*	0.04
10	1178	14.5	1.4	633	16.6	2.7	554	17.2	3.0	2.0*	0.07	0.6*	0.05
11	1312	15.0	1.6	584	17.2	3.0	584	17.5	2.9	2.2*	0.07	0.2	0.02
12	1340	15.6	1.9	579	17.9	3.0	734	18.4	3.3	2.3*	0.08	0.5*	0.04
13	1159	16.5	2.0	633	18.8	3.4	640	19.2	3.2	2.3*	0.08	0.5*	0.03
14	1152	17.4	2.1	617	19.5	3.0	667	19.6	3.0	2.1*	0.07	0.1	0.01
15	1275	18.0	2.0	552	19.7	2.6	637	19.8	3.1	1.7*	0.06	0.1	0.01
16	1267	18.4	2.0	520	20.0	2.8	590	20.4	3.4	1.6*	0.05	0.4*	0.03
17	984	18.6	1.9	536	20.3	2.7	642	20.3	3.6	1.7*	0.06	0	0
18	614	18.8	2.0	334	20.3	2.5	453	20.4	2.8	1.6*	0.05	0.1	0.01

*: p-value < 0.05

d1: Difference of mean between 1963 and 1993

d2: Difference of mean between 1993 and 2005/6

d1/yr: Annual changes between 1963 to 1993

d2/yr: Annual changes between 1993 to 2005/6

**Table 4:** Age-sex-specific body mass index (BMI) percentiles for Hong Kong in 1993 and 2005/6 and for mainland China in 2005 for children and adolescents aged 7 to 18 y [14]

Age (years)	Hong Kong 1963		Hong Kong 1993		Hong Kong 2005/6		China 2005	
				
	P_85_	P_95_	P_85_	P_95_	P_85_	P_95_	P_85_	P_95_
**Boys**								
7	15.1	16.1	17.9	19.6	18.8	21.4	17.0	19.6
8	15.5	16.7	18.4	20.3	19.4	22.2	17.7	20.4
9	15.5	16.6	19.1	21.2	20.1	23.1	18.4	21.3
10	15.9	17.1	19.7	22.2	20.9	24.1	19.4	22.2
11	16.6	18.1	20.3	23.0	21.6	25.0	20.1	23.0
12	17.2	18.9	20.8	23.7	22.3	25.7	21.0	23.9
13	18.0	19.8	21.3	24.4	22.7	26.2	21.9	25.0
14	18.5	20.2	21.9	25.1	23.1	26.6	22.8	25.9
15	19.2	20.9	22.3	25.6	23.5	26.9	23.5	26.8
16	19.9	21.7	22.8	26.1	23.7	27.2	24.2	27.7
17	20.3	22.1	23.2	26.4	23.9	27.3	25.0	28.5
18	20.5	22.2	23.4	26.6	24.1	27.3	24.8	28.2
								
**Girls**								
7	14.9	15.9	17.2	19.1	17.7	19.8	16.4	18.3
8	15.1	16.2	17.6	19.6	18.3	20.6	17.0	19.0
9	15.5	16.7	18.0	20.3	18.9	21.4	17.7	19.8
10	15.9	17.2	18.7	21.2	19.6	22.2	18.4	20.7
11	16.6	18.1	19.5	22.3	20.3	23.1	19.1	21.8
12	17.5	19.3	20.4	23.4	21.1	24.0	20.0	22.7
13	18.5	20.4	21.3	24.3	21.9	24.9	20.9	23.5
14	19.5	21.5	22.1	24.8	22.5	25.5	21.9	24.4
15	20.0	21.9	22.5	25.1	22.9	25.8	22.9	25.3
16	20.4	22.3	22.7	25.1	23.1	26.0	23.9	26.2
17	20.5	22.3	22.8	25.2	23.2	26.0	24.4	27.0
18	20.8	22.7	23.0	25.2	23.2	25.9	24.2	28.0

P_85_: 85^th ^percentile, overweight

P_95_: 95^th ^percentile, obesity

**Table 5:** Overweight and obesity prevalence by gender in Hong Kong children and adolescents using different cut-offs, for data from 1993 and 2006

	Boys		Girls		Total	
	1993	2005/6	1993	2005/6	1993	2005/6
			
**International Obesity Task Force cut-offs**[9]						
Overweight only (equivalent to adult BMI 25–30)	10.4%	15.8%	7.7%	10.1%	9.0%	13.0%
Obese only (equivalent to adult BMI >= 30)	3.4%	5.1%	1.8%	2.4%	2.6%	3.7%
Overweight and obesity (equivalent to adult BMI >= 25)	13.8%	20.9%	9.5%	13.5%	11.6%	16.7%
						
**Overweight and obese defined as >= 120% median weight-for-height**						
Total sample	12.0%	17.4%	9.4%	11.0%	10.7%	14.2%
Sub-sample of students reporting attendance to Student Health Service during previous year (n = 8199) (see text)	-	18.1%	-	10.7%	-	14.4%
Student Health Service estimate *	-	-	-	-	-	18.4%
						
**Percentiles based on 1993 data**						
**>= 85^th ^percentile**	16.7%	25.2%	16.7%	20.6%	16.7%	22.9%
**>= 95^th ^percentile**	7.7%	11.8%	6.6%	8.4%	7.1%	10.1%

* KH Mak, Student Health Service, Department of Health, Personal Communication

**Figure 1:**
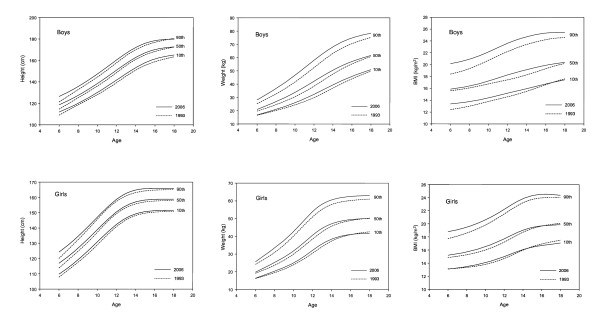
Comparison of height (cm), body weight (kg) and body mass index (BMI, kg/m^2^) for Hong Kong Chinese children and adolescents aged 6 to 18 y between 1993 and 2005/6.

**Figure 2:**
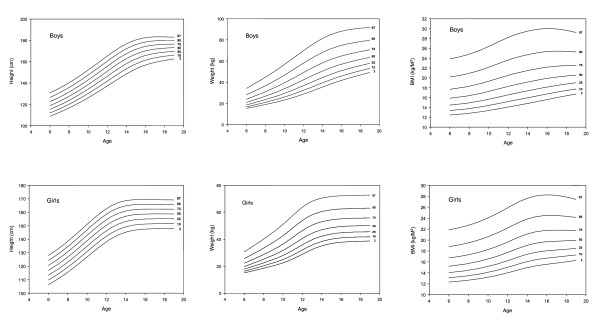
Percentile curves of height (cm), body weight (kg) and body mass index (BMI, kg/m^2^) for Hong Kong Chinese children and adolescents aged 6 to 18 y for 2005/6.

The percentile curves for height, weight and BMI developed using the LMS method were compared for the 1993 and 2005/6 data sets (Figure 1). Percentile curves (3^rd^, 10^th^, 25^th^, 50^th^, 75^th^, 90^th^, 97^th^) for height, weight and BMI were developed for the 2005/6 data (Figure 2).

### Secular changes in height

**Figure 3:**
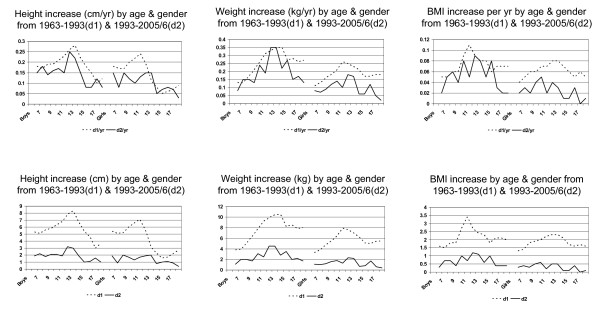
Total and annual difference of mean between 1963 and 1993 (d1) and between 1993 and 2005/6 (d2) by gender for height (cm), body weight (kg) and body mass index (BMI, kg/m^2^) for Hong Kong Chinese children and adolescents aged 6 to 18 y.

For each age group and for both genders, height increased significantly both from 1963 to 1993 and again from 1993 to 2005/6 (Table 1). During both periods, the height increases were greater in the younger children, and particularly in the boys, who were 3 cm taller at ages 12 and 13 y, while girls' maximum height differences were 2 cm at age 8 y and again at ages 12 and 13 y. To better compare changes over the two different time periods, 1963 to 1993 and 1993 to 2005/6, the annual increases in height were also calculated (Table 1, Figure 3).

### Secular changes in weight and BMI

For all age and gender groups, weight and BMI increased from 1963 to 1993 and again from 1993 to 2005/6 (Tables 2 and 3, Figure 3). However for the girls older than 13 y, neither the weight nor the BMI increases were significant in the latter period. For boys, however, although the weight increased significantly at all ages during the latter period, BMI increased significantly only up through age 15 y during that time. Again, therefore as for height, during both periods, the weight and BMI increases were greater in the younger children, and particularly in the boys. The annual increases in weight and BMI tended to be greater in the earlier period, 1963 to 1993, than in the latter period, 1993 to 2005/6 (Tables 2, 3).

### Increase in overweight and obesity

The 1993 and 2005/6 datasets were analysed according to different cut-offs to define overweight and obesity to reveal the percentages of overweight or obese children and adolescents in those two surveys (Table 5) [7,9]. Further detailed comparisons of BMI by age group were made with recently published data from mainland China, which showed similar proportions of children having BMI above the 85^th ^and 95^th ^centiles (Table 4) [14]. Of the sub-sample of 8199 students who had attended the SHS during the current or previous year, 14.4% were >= 120% median weight-for-height, the SHS criteria to define obesity (Table 5).

## Discussion

Our 2005/6 data collected from a large representative sample of Hong Kong children show that secular changes in linear growth continue, but of concern is the steady increase in the prevalence of overweight and obesity. Although the International Obesity Task Force (IOTF) cut-offs for overweight and obesity are useful for international comparisons, and comparisons over time, these cut-offs do not necessarily equate to children at risk of the complications of obesity. In April 2006, WHO launched new Child Growth Standards (CGS) based on data from 8440 children in six countries raised in environments that promote healthy growth (breastfeeding, good diets, prevention of infections and healthy mothers who do not smoke) [15]. Recognising that using descriptive data from populations that reflect secular trends towards overweight and obesity to construct growth references will inadvertently result in upward skewness that will lead to an under-estimation of overweight and obesity and an over-estimation of under-nutrition, WHO has also constructed and recommended new growth references for school-aged children and adolescents aged 5 to 19 y [16,17]. These new references accord with the new pre-school WHO CGS and BMI cut-offs for adults.

Although our estimates of the prevalence of overweight and obesity varied when different criteria were used, the gender differences persisted irrespective of the cut-off (Table 5). Using the IOTF cut-offs, 16.7% of Hong Kong children (20.9% in boys and 13.5% in girls) were overweight or obese and 3.7% were obese in 2005/6, while in 1993 the figures were 11.6% (13.8% in boys and 9.5% in girls) and 2.6%, respectively [9]. Using the definition of "weight >= 120% of median weight-for-height", which is the measure used by the Hong Kong SHS, 14.2% of children were overweight/obese in 2005/6, compared to 10.7% in 1993. However routine data collected by the SHS for 2005/6 showed that 18.4% of school children were classified as overweight/obese based on this definition (KH Mak, Student Health Service, Department of Health, Personal Communication, Table 5). This discrepancy implies a systematic bias in either or both samples, and has important implications for using the SHS data to monitor the obesity epidemic. Although 65% of our sample who responded to the question "whether they had attended SHS for physical examination in the previous year" was similar to the SHS estimate of 56–58% for 2004/5, the proportion of primary attendees was less (65% versus 80%) and the secondary attendees was more (64% versus 30%). Also it was possible that students may have misinterpreted the question on whether they had attended the SHS for physical examination in the current or previous year. Although all SHS attendees have their weight and height measured annually, they will only have a detailed physical examination by a doctor every second year. Further study is therefore warranted to establish whether normal weight students are less likely to attend the SHS, or whether overweight children are less likely to enrol in a school-based study such as ours.

There is no universally agreed definition of overweight and obesity [18]. However despite limitations, BMI is considered a valid and feasible indirect measure of body fatness. The IOTF BMI cut-offs provide a common set of references that help to make international comparisons [18]. For example recent IOTF data suggest that between 1.6% (in Africa) and 28% (in the Americas) of children are overweight and 0.2% (in Africa) and 9.6% (in the Americas) are obese. It has been projected that the prevalence of overweight and obesity for 2010 will increase to 46% and 15% (in the Americas) and 23% and 5.3% (in South East Asia), respectively [17]. Although the prevalence in Hong Kong may not be as high as in some other countries, the approximate 0.5 percentage point rate of annual increase in the prevalence over the past decade is similar to that of countries such as the United States, which is a cause for great concern. Ethnic differences also need to be considered when interpreting data, since Asian adults appear to experience the adverse effects of overweight and obesity at lower BMI cut-offs than do Caucasians [19]. However, even within different Asian populations there are significant differences in size and morbidity risk relationships so it has been difficult to define BMI cut-points that apply to all Asian populations for predicting increased health risks [20].

Our data suggests that the proportional increase in overweight and obesity has been greatest in the youngest children (Table 3). Three possible explanations for this greater difference in younger children can be considered. First, it is possible that children are experiencing the adiposity rebound at a younger age, but then they level off and are not significantly more overweight or obese than they were in 1993 in the older age groups. Second, children may be starting puberty earlier, although it is controversial whether there is any causal relationship between early maturation and obesity [21]. Third, we may be witnessing the effect of an advancing wave of the obesity epidemic. Younger children in our present cohort, that are taller and heavier than similar aged children in 1993, may reflect the evolving progress of the obesity epidemic, and that this particular cohort of children may continue to grow taller and become heavier than current children in the older adolescent age group. Longitudinal surveys or repeated cross-sectional surveys with puberty assessment could help to clarify this further.

WHO has recommended that Ministries of Health, national paediatric associations and other policy makers need to decide whether or not to officially adopt the WHO CGS . Our study did not include the pre-school age group but we will still need to consider for the 5 to 19 y age group whether Hong Kong should continues to use our current 1993 growth reference charts, whether we should update these charts with our 2005/6 data given the secular trends noted or whether we should adopt the new WHO 5 to 19 y charts. More detailed comparisons and discussions with the various stakeholders will be required to make this decision. Meanwhile, our data provides policy-makers with further hard evidence to emphasise the urgency of the problem of increasing obesity. Although the economic implications of the obesity epidemic are still to be fully quantified [22], it is likely that investment in prevention strategies targeting different societal levels now may prevent not only future avoidable death and disability, but will also be cost effective in the longer term.

## Conclusion

Going forward, further studies and regular surveillance of the growth of Hong Kong children are required, both to understand how the obesity epidemic will continue to unfold, as well as to assess the impact of interventions instituted.

## Abbreviations

BMI: Body mass index; IOTF: International Obesity Task Force; MCHC: Maternal and Child Health Centres; SHS: Student Health Service; WHO: World Health Organization.

## Competing interests

The authors declare that they have no competing interests.

## Authors' contributions

HKS coordinated the study, assisted in the supervision of data collection, and took active part in the statistical work. EASN, RYTS and AML prepared the proposal and supervised the study. EMCW and JTFL provided advice in data analysis. GSS, KHM, YW and TFF made substantial contributions to the conception of the study and revising the manuscript. All authors read and approved the final manuscript.

## Pre-publication history

The pre-publication history for this paper can be accessed here:


